# Autonomy, power dynamics and antibiotic use in primary healthcare: A qualitative study

**DOI:** 10.1371/journal.pone.0244432

**Published:** 2020-12-18

**Authors:** Laura Medina-Perucha, Ana García-Sangenís, Ana Moragas, Pablo Gálvez-Hernández, Josep María Cots, Anna Lanau-Roig, Alícia Borràs, Isabel Amo, Ramon Monfà, Carl Llor, Anna Berenguera

**Affiliations:** 1 Fundació Institut Universitari per a la recerca a l'Atenció Primària de Salut Jordi Gol i Gurina (IDIAPJGol), Barcelona, Spain; 2 Universitat Autònoma de Barcelona, Bellaterra (Cerdanyola del Vallès), Spain; 3 Universitat Rovira i Virgili, Jaume I Health Centre, Institut Català de la Salut, Tarragona, Spain; 4 Institut Universitari de Pacients (Patients’ University Institut), Universitat Internacional de Catalunya, Barcelona, Spain; 5 University of Toronto, IHPME-BFON Collaborative PhD Specialization Program in Health Services and Policy Research, Toronto, Ontario, Canada; 6 La Marina Health Centre, Institut Català de la Salut, Associació d’Infermeria Familiar i Comunitària de Catalunya, Barcelona, Spain; 7 Escola Universitària d'Infermeria, Escoles Universitàries Gimbernat, Universitat Autònoma de Barcelona, Sant Cugat del Vallès, Spain; 8 Via Roma Health Centre, Institut Català de la Salut, Barcelona, Spain; 9 Departament d'Infermeria, Universitat de Girona, Girona, Spain; City, University of London, UNITED KINGDOM

## Abstract

Antibiotic resistance is a global health concern. Although numerous strategies have tried to reduce inadequate antibiotic prescribing, antibiotics are still prescribed in 60% of acute lower respiratory tract infections (ALRTIs) cases in Catalonia (Spain). This study aims to explore service users’ experiences of ALRTIs, the quality and access to healthcare services, and health education. Selective purposive sampling was carried out, based on a prior definition of participant characteristics. These were sex, age, ethnicity, date of the last ALRTI, number of ALRTIs in the last year, and treatments received. Participants with a previous diagnosis of ALRTIs were recruited from three primary health care centres in Barcelona and one in Tarragona. Twenty-nine interviews were conducted between April and June 2019. A content thematic analysis was performed. Three themes were identified: 1) risk perceptions and help-seeking; 2) treatment preferences and antibiotic use; and 3) relationship dynamics and communication with healthcare professionals. Accounts of service users’ sense of autonomy towards their health and power dynamics within the healthcare system were apparent. Supporting service users to become reliable, subjective and agentic experts of their health and bodies could help them to voice their healthcare agendas. Power structures embedded within healthcare, political and economic institutions should be challenged so that healthcare services can be co-developed (with service users) and based on service users’ autonomy and horizontal relationships. Special consideration should be paid to the intersection of social vulnerabilities. A concordance approach to prescribing could be key to improve the responsible use of antibiotics and to contribute to the prevention of AMR in primary healthcare. The marketisation of health, and the increased demands of private healthcare in Spain due to the financial pressures on public healthcare as a consequence of the financial crisis of 2008 and the COVID-19 pandemic, are a risk for promoting adequate antibiotic prescribing and use.

**Trial registration** The ISAAC-CAT study has been registered in the NCT registry, ID: NCT03931577.

## Introduction

Antimicrobial resistance (AMR) occurs when microorganisms (e.g., bacteria or viruses) change as a consequence of being exposed to antimicrobial drugs such as antibiotics. Some of these microorganisms develop resistance towards medicines that become no longer effective in treating a certain condition (e.g., an infection). AMR is a major concern worldwide as it compromises the ability to treat even common infectious diseases, as well as it increases morbidity, disability and mortality [1–4]. AMR also raises healthcare costs [4] and may endanger the achievement of the Sustainable Development Goals [5] (e.g., Goal 3: ensure healthy lives and promote wellbeing for all at all ages). In 2015 the World Health Organization (WHO) prepared a global action strategy to address AMR [2]. This strategy focused on the importance of involving entire societies, prevention, equitable access to (and appropriate use) of new antimicrobial medicines, a sustainable use of resources, and promoting incremental targets for implementation of AMR strategies [2].

Overuse and misuse of antibiotics have contributed significantly to the development of AMR [1]. Most antibiotic prescriptions are processed in primary healthcare services, most commonly to treat acute lower respiratory tract infections (ALRTIs) [6–11]. Even if recent research has highlighted the reduced benefit of prescribing antibiotics [12, 13], 60% of ARLTIs cases are still treated with antibiotics in Catalonia (Spain) [14]. Based on a report of the Spanish Agency of Medicines and Medical Devises, the overall antibiotic intake was over 32 defined doses per 1,000 daily, one of the highest consumption rates in Europe [15]. The high intake rates in Spain have also been recorded by European agencies [16, 17].

A crucial element to reduce antibiotic prescription (and use) is to increase awareness and educate both society and healthcare professionals on AMR [2]. However, available evidence suggests that educational interventions may only generate small changes in prescription practices, and that multi-faceted approaches may be more effective than single-component interventions [18, 19]. As an example, the combination of C-reactive protein (CPR) rapid testing, shared decision-making, and procalcitonin-guided management was successful in a study to reduce unnecessary antibiotic prescribing [20]. Other effective interventions have focused on accountable justification and peer comparison [8].

Service users’ expectations and satisfaction with healthcare consultations are essential to reduce service users’ demands and the use of antibiotics, especially considering the frequency of the use of non-prescribed antibiotics worldwide [21, 22]. Lack of knowledge and beliefs around antibiotic use and AMR among the public [23] and healthcare professionals [24, 25] could be contributing to the increased use of antibiotics. Communication between antibiotic prescribers and users is a key element to tackle antibiotic overuse [26–29]. As already highlighted in the literature, communication should focus on adequate provision of information [26], the de-medicalisation of self-limiting infections [27], promoting users’ empowerment [28] and facilitate shared-decision making [26, 30, 31].

Recent qualitative research on this area has explored service users’ [32] and prescribers’ [33–37] attitudes towards delayed antibiotic prescription, prescribers’ attitudes and experiences on antibiotic prescribing [38–54], service users’ experiences on antibiotic use [38, 39, 48, 51, 55–58], self-care treatments for ALRTIs [32, 59], service users’ [48, 60] and health professionals’ [47, 48] understandings of AMR and antibiotic use, interactions between health professionals and service users [47], experiences of ALRTIs [59], the role of pharmaceutical companies on antibiotic overprescribing and AMR [39], and socio-cultural factors to promote adequate antibiotic use [61]. These studies are mostly focused on health professionals’ experiences (rather than service users’) and take a conventional biomedical perspective to health and AMR. Despite the vast amount of research on AMR and antibiotic prescribing/use worldwide, qualitative research in this topic in Spain is rather scant. The present study aimed at addressing these gaps, by using qualitative research to explore the experiences and concerns of service users with ALRTIs, in relation to access to healthcare, antibiotic use and health education in Catalonia (Spain).

The qualitative research presented in this article is part of the ISAAC-CAT study [62], a project that includes a randomised controlled trial (RCT) that aims to compare three interventions (clinician disease-focused intervention; continuous training; treatment as usual) to reduce antibiotic prescribing for people with an ALRTI, in primary healthcare settings in Catalonia (Spain). A pre-intervention qualitative study (the research presented in this article) was designed to inform the development of the RCT, and to give an insight on socio-structural accounts to promote a responsible use of antibiotics and prevent AMR.

## Materials and methods

### Design

A qualitative study was a relevant approach to explore the experiences of ALRTIs and healthcare in primary care settings among people who had a previous ALRTI diagnosis. Semi-structured interviews were conducted in their own words and through their own interpretations of their realities [63].

### Participants and setting

Participants were adults diagnosed with an ALRTI in the last six months. Several characteristics were considered to ensure diversity of experiences and accounts: participants’ age, sex, ethnicity, date of last ALRTI, number of ALRTI in the last year, and ALRTI treatments received. Participants were primary healthcare service users and were recruited from four primary healthcare centres, three in Barcelona and one in Tarragona (Catalonia, Spain).

### Participant recruitment

Recruitment was selective and purposive. There were several stages of recruitment. First, eligible participants were identified by healthcare professionals working at participating in primary healthcare centres. The recruiters were commonly (but not always) the participants’ doctors or nurses. These potential participants were then approached by healthcare professionals and gave information about the study. Contact details of those individuals that expressed interest in participating were then shared with the research team, with the potential participants’ consent. Forty-one people were contacted by one of the researchers (LMP) and 29 participated in individual semi-structured interviews. Participants were selected to ensure diversity in participants’ accounts, based on their sex, age, ethnicity, date of the last ALRTI, number of ALRTIs in the last year, and treatment regimens received. Data were collected until saturation was reached.

### Materials

A topic guide was developed for the individual semi-structured interviews. This was developed by all researchers participating in the ISAAC-CAT study.

The topic guide included: 1) experiences of ALRTIs; 2) conceptualisation of symptoms and language used; 3) red flag symptoms and reasons for healthcare consultations; 4) healthcare services attended for ALRTIs; 5) treatment experiences; 6) preferences and types of treatments; 7) knowledge of antibiotic resistance; 8) healthcare professionals’ communication skills; 9) preferences and needs for written materials on ALRTIs. The topic guide in Spanish and an English translation are available as appendices to this article.

### Procedure

All interviews were conducted in primary healthcare centres. First, participants were given the study information sheet and were allowed the time and space to ask questions and resolve any doubts. Verbal and written consent was required from all participants. Individual interviews took between 21 and 84 minutes and were conducted by LMP (n = 24) and AB (n = 5). The researchers (LMP, AB) adjusted the timings of the interviews based on the participants’ needs. All participants were also asked to complete a non-standardised questionnaire to collect sociodemographic data. The interviews were conducted in Spanish and Catalan, based on the participants’ preferences.

The interviews were audio-recorded. One interview participant preferred not to be audio-recorded as it caused her distress, given that she had speech difficulties. In this case, the researcher took notes during the interview with the verbal consent of the participant. The recorded interviews were transcribed verbatim by external transcribers and one of the researchers.

### Data analysis

Thematic Content Analysis was used to analyse the interviews’ data, as described in Berenguera et al. [64]. Data were analysed following these steps: 1) pre-analytical insights through successive readings of the transcribed interviews; 2) identification of relevant themes; 3) fragmentation of the text into units of meaning; 4) text codification, with emerging codes from the data; 5) creation of categories, by grouping the codes based on the criterion of similarity; 6) analysis of each category; and 7) elaboration of new text based on the findings.

Triangulation was performed to ensure the quality and richness of the qualitative analyses [65, 66]. Three interviews were analysed independently by two researchers (LMP, AB). Analyses were then discussed between the two researchers first until consensus was reached. These were then presented and discussed with other members of the research team. Findings were then discussed until any disagreements were resolved.

### Ethics

This research received ethical approval by the Research Ethics Committee of the Institut de Recerca en Atenció Primària Jordi Gol i Gurina (IDIAPJGol) (REC reference P18/227, 19^th^ December 2018). The study has been conducted based on to the Declaration of Helsinki and Good Clinical Research Practice.

All participants were given information about the study by a healthcare professional and the researchers prior to their participation. They were also given the time to resolve any doubts. Verbal and written consent was obtained before the interviews were initiated. A pseudonym has been chosen for each participant to ensure anonymity and confidentiality. Participants gave consent for their anonymised data to be shared. All data were stored securely and are only accessible to the research team.

## Results

### Participant characteristics

Twenty-nine primary healthcare service users were interviewed (16 women; 13 men). They were between 25 and 89 years old (*M* = 57). All participants were White, except for one that was Latino. Most people were from Spain (n = 26). Some participants were working full-time (n = 12) at the time of the interviews. Other were retired (n = 8), on health benefits (n = 4), unemployed (n = 1), homemaker (n = 1), or on full-time education (n = 1). Only one participant was a full-time student and two people were studying during their retirement. Participants had completed primary education (n = 9), high-school (n = 6), professional training (n = 2), university-entry studies (n = 4) a university degree (n = 7), and a postgraduate degree (n = 1). They were service users of four primary healthcare centres in Barcelona.

### Main findings

Three themes were identified: 1) risk perceptions and help-seeking; 2) treatment preferences and antibiotic use; and 3) relationship dynamics and communication with healthcare professionals.

### Risk perceptions and help-seeking

Even if participants would not commonly worry about particular symptoms, they would often express concerns about their symptoms being a sign of another “more serious” health condition, such as cancer.

*“I know*. *As I said*, *I hope that it is nothing serious*. *They would have detected something if I had*… *I think*, *I don’t know [*…*] I wonder if the cancer is there and I do not realise (laughs)*… *I don’t know*… *(P29*, *woman*, *55 years old*).

Participants discussed what they thought were risk factors for them to become ill. Some of these factors were attributed to lifestyle decisions, such as smoking, obesity, diet or stress. External factors were also mentioned, such as the weather and transmission from children. Others also considered immigration and poverty as health risk factors, related to neighbourhood unsafety. A few participants even explained how they changed some of their day-to-day activities or being concerned to go outside to the street. In some cases, participants held stigmatising and discriminatory attitudes towards migrants, ethnically minority populations (e.g., Roma community) or socio-economically deprived populations. On the views of these participants, these vulnerable groups are to blame for a heightened risk of disease transmission.

*“Everyone who gets into* [the country], *they should get in healthy*. *Sure*, *because it is a danger to the whole population (…)*. *So this is misery*, *of people who live in misery”*. *(P25*, *man*, *66 years old*).

Participants did not usually mention “red flag” symptoms. Still, they would seek professional help based on their constructions of health, subjective wellbeing and self-perception of impairment on their day-to-day activities and responsibilities. Help-seeking was then triggered when each person considered that the duration and severity of the symptoms required healthcare advice and treatment and when their health condition was impairing their daily life. The concept of “impairment to daily life” was subjective and different for each person, although it was commonly associated with not being able to fulfil their responsibilities for what each considered an acceptable amount of time.

*“No*, *is when I really feel I can’t*. *If I can endure it then I do not go*. *But if I see that*… *that I cannot*… *then I come*. *This last time that I am telling the* [doctor], *is that (inhales deeply) is because I was saying ‘something’s gonna happen to me’*. *But oh well*…*” (P2*, *woman*, *73 years old*).

Participants (and particularly women) who had children or other people to care for, and people who were working full-time (especially those who were self-employed) often mentioned delaying help-seeking as they struggled to find time to access healthcare services and practice self-care to recover. In some cases, participants only attended healthcare services when the ARLTI was severe, and they felt a sense of urgency. In some of these cases, participants had to access emergency services or get hospitalised.

Attending healthcare services was linked to a need for certainty and a “quick fix”. Consultations with healthcare professionals were mostly framed around the need for a solution that participants could not find themselves. Visits to healthcare services were also prompted when they had previous negative/unsatisfactory experiences with other healthcare professionals (e.g., attending the emergency unit if they had a negative or unsatisfactory experience with their family doctor). Re-visits were also arranged when, even if the symptoms had been cleared, there were still residual symptoms (such as cough).

People with comorbidities mentioned to access healthcare services quick as they felt more urgency, compared to other people. Getting a healthcare consultation was useful to reduce uncertainty around their health condition, especially when there were comorbidities, and to get more certainty on when they will recover.

Primary healthcare centres were the most mentioned and trusted settings to access healthcare. Other common settings to access were community pharmacies, hospitals and alternative medicine practitioners. Participants accessed hospitals when they had to attend the emergency room or were referred by a primary healthcare professional to a specialist (e.g., pulmonologist).

*“Sometimes it is what I telling you*, *I have a cold*, *I come to the emergency services*, *I know what I have*, [family doctor] *knows what I have*, *and sometimes they* [staff at emergency services] *don’t even listen to you*.*” (P18*, *man*, *60 years old*).

Accessing private healthcare services was an option that some participants mentioned. Although these services were not perceived as trusting and of good quality compared to public healthcare services, they were more reliable in responding to service users’ demands (e.g., of getting a prescription for antibiotics). According to participants, public healthcare services were better for “severe” health conditions but private healthcare services often offered quicker and more satisfying services (based on service users’ expectations).

*“If you have a profession that limit many times your diary*, *and obviously it is not a very severe health condition*, *then*, *all this*… *here there is an advantage* [quick services] *of private healthcare over the public one” (P24*, *man*, *56 years old*).

Participants normally expected and preferred to get a consultation with a doctor, rather than a nurse. The role of nurses was perceived to be for non-urgent visits, lifestyle habits and follow-up appointments (e.g., to lose weight). The preference of a doctor was also based on the fact that nurses cannot prescribe medication and, when participants felt unwell, they expected to get a prescription. Thus, getting visits with a nurse often created distrust among service users.

Time in healthcare services was one of the topics discussed in relation to help-seeking and access to healthcare. Participants expressed how they usually had to wait (what they considered to be) a long time to get a visit scheduled with their family doctor or nurse. Also, when they attended these appointments they would usually have to wait as there were often delays in healthcare services. Although some participants complaint about healthcare professionals for not managing their time well, most blamed the healthcare system for restricting consultation times to only a few minutes when many visits required more time. Even if they had to wait on the day of the appointment, participants were understanding and empathetic towards other service users needing more time for their consultations, and towards healthcare professionals for “doing what they could”. Some participants expressed how they had to attend emergency services, despite these were overloaded, as opposed to primary healthcare services as there could not get an appointment quickly enough when they needed it.

*“*[If] *I am feeling unwell*, *well*, *I can’t book an appointment in three days’ time*. *And what I can’t do is contributing to the overload in emergency services*. *I think that we have a deficiency here”*. *(P24*, *man*, *56 years old*).

There were some cultural differences in the expectations held on the healthcare consultation and the need for treatment among participants. These are important to consider for healthcare professionals to know how to communicate and manage service users’ expectations.

*“We should take antibiotics*, *a day passes by and the pain and everything else are there again […] I say no*, *nobody will help me here*. *I booked my flights and I went to my country because I knew that there was one* [doctor] *there*… [who would prescribe her antibiotics].*” (P11*, *woman*, *35 years old*).

#### Treatment preferences and antibiotic use

Most participants referred to themselves as being “anti-medication”. For most, this meant that they would prefer not to take medication; some would prefer natural remedies, but others would rather not use medication and wait to recover naturally. This however changed when participants considered they were *too* unwell, or had been unwell for a long *enough* time. In these situations, participants felt a sense of urgency and wanted quick solutions. This was usually when they sought help to healthcare professionals and accessed healthcare services. Participants expected to get a “fast and definite solution” from healthcare professionals to recover their health as quick as possible and, often, without much effort.

Some participants used natural remedies, such as steam inhalations, infusions, cough sweets and lozenges and honey. A few participants also mentioned eating oranges and lemons to boost the immune system and as a preventive measure. Some people did not use natural remedies, although this was not common.

*“Normally yes*, *yes because they are very basic and they are useful for a basic condition*. *Well*, *if you have a severe condition then go to the doctor* […] *and maybe they* [natural remedies] *are more beneficial than getting drugs” (P24*, *man*, *56 years old*).

Most people were self-medicating with analgesics and over-the-counter medicines. Despite antibiotics are not legally dispensed without a prescription, a few participants mentioned self-medicating with antibiotics. For any medication, if participants felt that they were not getting any benefits from taking it or they felt recovered, they would stop using it.

When accessing healthcare services, participants expected to get medication, ideally one that could improve their health condition within a short period of time. If this expectation was not met (e.g., they were not prescribed a medicine), participants would often feel frustrated. Faced without the solutions that they were looking for, participants explained how they sometimes scheduled another visit to the same or another healthcare professional. Other times they would follow the healthcare professional’s advice from that first visit. Participants did not express demanding an antibiotics prescription generally. Still, they thought that having a prescription for antibiotics decreased the uncertainty regarding the ARLTI, especially when they had been unwell for a *long* time.

In some occasions, participants expressed frustration when they expected a prescription for antibiotics and they did not get it when they accessed healthcare services. Participants explained that they sometimes felt certain that they had an infection and so they needed antibiotics. Interestingly, when they recovered without antibiotics they felt relieved that the healthcare professional did not prescribe antibiotics, as they preferred not to take them. On the other hand, participants would get upset if they had to re-visit and got antibiotics prescribed on a follow-up visit. In these situations, they felt that they had been losing their time re-visiting, their symptoms had worsened and it was taking them longer to recover. There was also a negative impact on the trust placed on that healthcare professional and even health services as a whole.

*“You start distrusting*, *not losing trust*, *but when they prescribe something and it does not solve the problem*, *you go back and… it doesn’t solve you the problem*, *and you go back and it doesn’t solve you the problem… and then you end up thinking if they know enough to solve the problem […] it is when you end up*, *somehow*, *losing trust in the doctor*.*” (P21*, *man*, *71 years old*).

Participants perceived antibiotics as a type of medication that was mainly prescribed when there was an infection that was somehow severe. Antibiotics were also perceived as being very effective. So, for many, having a prescription for antibiotics meant a speedy recovery.

A few participants mentioned having some knowledge on antibiotic resistance, although this was not common.

*“*[Her daughter] *went to pay* [private healthcare services], *they gave her antibiotics*, *yes but*, *so what*? *The day that your daughter needs an antibiotic for real*, *maybe it’s not going to be effective*, *because if she has a cold often and you keep giving her antibiotics*. *Things* [recovery] *need their time*.*” (P16*, *woman*, *65 years old*).

Sources of information on antibiotic resistance were mostly the media, personal (or other people’s) experiences, or through formal education. Healthcare professionals were not mentioned as a source of knowledge. Knowing about antibiotic resistance did not necessarily mean that they would not misuse antibiotics. One participant had a healthcare degree and worked full-time as a carer (P29; woman, 55 years old). Despite her knowledge and knowing that it was not a good decision, she mentioned taking antibiotics without a prescription:

*“Sometimes when I have Amoxicillin or something*, *I just take it*, *because I say*, *I have*, *I have*, *I have taken it before*, *so I take it” (P29*, *woman*, *55 years old*).

There were other few cases of participants who had taken non-prescribed antibiotics, but this was not common. In few occasions, participants mentioned having gotten antibiotics from their community pharmacy without a prescription and taken them, or use leftover antibiotics that they had at home. Others had done it in the past but they were not doing it anymore, seemingly because of their increased awareness on the importance to use antibiotics correctly.

*“A few times they have given me some pills* [antibiotics] *for something*, *if it has happened again after some time*, *then instead of coming*, *I had taken the medication directly” (P19*, *man*, *48 years old*).

Some participants mentioned that they had stopped taking the course of antibiotics as soon as they were not feeling unwell anymore. One participant mentioned having had a personal experience on antibiotic resistance (*P17*, *woman*, *71 years old*).

Overall, participants mentioned that they feel that doctors are not very keen on prescribing medication, including antibiotics.

#### Relationship dynamics and communication with healthcare professionals

Vertical relationships were identified between healthcare professionals and service users, being healthcare professionals where the power in the relationship resided. Healthcare professionals were “giving” information to *their patients*. The later expressed their need for a more horizontal and dialogic relationship.

*“I come*, *he tells me off*, *and I just fawn…" (P19*, *man*, *48 years old*).

Some participants felt (and worried) about “complaining” to healthcare professionals and feeling less valued by them for this reason. They sometimes had a feeling of disempowerment and felt they were “below” healthcare professionals, a barrier that deterred them from asking questions for example. In line with this, it was mentioned that it was important not to visit healthcare professionals many times, neither being “too demanding” in order to have a “good” relationships with healthcare professionals.

*“I took the opportunity of being in the emergency room* [for her daughter] *to ask the doctor to also see me*, *because I was ashamed of going back here* [primary healthcare centre] *if I had come the day before […]*. [Interviewer: What is making you feel ashamed?] *Coming back here*, *because I had come a couple of times… and I don’t know*, *I don’t know*. *In reality… I am ashamed*, *it feels as if you need to convince them of something all the time […]*. *But then I had to come back on Tuesday because I had gotten worse”*. *(P8*, *woman*, *39 years old*).

Most participants generally followed healthcare professionals’ indications. However, it seemed that there were tensions between the ones that thought that healthcare professionals (because of their knowledge) were the responsible for their own (service users) health, and the ones that needed to feel in control of their own body and health.

*“But it depends*, *apart from what the doctor tells me*, *you also know your own body*, *and if you feel weak*, *don’t screw it up and keep taking it* [the medicine].*” (P27*, *man*, *28 years old*).

However, based on participants’ accounts, decisions did not have to be made by healthcare professionals. Some (but not all) service users expected to be able to make their own decisions to care for their health and bodies. Healthcare professionals were seen as the experts in medicine or nursing, but service users were often seen as the experts on their own health and bodies. Thus, healthcare decisions had to be informed and shared with healthcare professionals. It was important for some participants that this was respected and understood by healthcare professionals, although this was not the norm.

*“I am not a fan of ‘oh*, *whatever you say’*. *No*, *I say this*, *and this and that*. *That’s all*. *I am the first one who’s interested because it is my body that they are treating with […]” (P25*, *man*, *66 years old*).

There was some tension between participants’ need for trust and certainty and their need for autonomy in relation to their own health. Participants explained how important trust in healthcare professionals and the healthcare system was for them. This was somehow related to the search for certainty in visits with healthcare professionals. Getting a “solution” from healthcare professionals to improve health led participants develop trust in healthcare professionals. These solutions translated into certainty around one’s health. At the same time, certainty and trust were sometimes perceived as incompatible with being autonomous to care for and make decisions about one’s health and body.

*“The doctor decides*, *he tells me what to do*, *then I am responsible of what I do*, *if I do it well or I don’t do it*.*” (P23*, *man*, *69 years old*).

Participants communicated their symptoms and signs from their subjective experience. However, this subjective communication seemed to sometimes clash with the apparent need for objectivity of healthcare professionals. These discrepancies made participants feel vulnerable and unheard sometimes, especially when they did not know how to manage their health and access healthcare services “to demand a solution” to healthcare professionals. Good communication, trust empathy-based interactions were thought to be crucial. These elements were especially important for people with comorbidities or mental health issues as these groups of service users seemed to feel especially vulnerable, and so they highlighted the importance of feeling heard and for their concerns to be understood.

*“When I’m like this* [having breathing difficulties] *I’m worried because the heart also suffers […]* [This participant was diagnosed with heart disease] *I was worried that it was going to have an impact on the heart*, *otherwise I wouldn’t have gone to the emergency room […] Sometimes you wish that the person that tells you that* [that there is nothing wrong with him and gave him no treatment]… *you wish that they spend seven or eight nights like the ones that I have spent a month almost*, *ah*? *That they go to bed and they can’t sleep” (P1*, *man*, *75 years old*)

Communication with healthcare professionals was generally thought to be insufficient. However, on some occasions, participants had clear and complete information on the aetiology, progress, preventive measures and treatment, methods to alleviate symptoms, duration for recovery, among other details. Despite some users expected more information on their health, they failed to obtain a desired response from healthcare professionals or had not asked any questions. Even though some participants experienced a lack information on ARLTIs, a few expressed not needing to know more.

Furthermore, participants also mentioned how healthcare professionals often did not share what procedures they would conduct *on* service users. This generated uncertainty and anger in many cases. While some people felt empowered to demand more information, others felt disempowered in these situations. Overall, participants claimed the need for information to be shared so that they could know what was being decided *for them* to be able to make their own decisions.

*“They prescribe you without listening to you […] I mean*, *she goes fast*, *whatever it is*, *oh this*, *or she understands herself*, *she knows… only with a glance she know what I have… I don’t know… […] Yes*, *I think they are not open to a dialogue*, *some doctors*.*” (P28*, *man*, *89 years old*).

As an example, one participant who was hospitalised for pneumonia explained how she believed she had cancer as none of the healthcare professionals (primary and secondary care) explained to her the reason for medical tests and procedures, referrals and hospitalisation. She was too scared (thinking that she had cancer) to ask, and because she would blame her poor health on herself (P13).

*“Because I was scared*, *I was scared*. *I thought that it was*, *it does not mean that I never get it*, *but a neo* [neoplasm] *in my lungs*, *because I was convinced*, *I don’t know*, *because my dad died young*, *I had been a smoker*, *I haven’t taken care of myself because… I don’t know if I can say it but I had a tough adolescence of one of my daughters*, *I mean*, *very distressing*. *And because I had a haemorrhage and I thought*, *I had not cared for myself*, *I have always gone… well*, *also smoking is my fault*, *not the fault of my daughters’ adolescence” (P13*, *woman*, *61 years old*).

Participants highlighted that it was important that their subjective experiences were considered. For them, their health was not just about objective signs of illness or health, but about the person’s own experience. Also, how one’s health condition and lived experience had an impact on their daily life, mood, social relationships, professional life, and so on. It was essential for participants for healthcare professionals to bear in mind this dimension to prevent re-visits, to improve quality of life and the duration/severity of symptomatology.

*“Of course*, *it is the fact*, *well*, *I think*, *that Medicine is for that*. *Medicine*, *who suffers*? *The patient*. *Then it is listening to the patient*, *their explanations*, *what happens to them*, *what doesn’t happen to them*.*” (P18*, *man*, *60 years old*).

Some people explained how they felt unheard by healthcare professionals. In such cases, participants often had to insist and convince healthcare professionals to trust their own perception of health and illness.

*“*[The doctor] *of the stomach*, *or endocrinologist*, *well*, *I don’t know who the hell it was*… *and he was saying ‘no*, *you are a young guy*, *it’s nothing’*. *I said ‘dad* [the participant’s dad was a doctor], *my stomach hurts for real and this is not normal*, *something’s happening to me*, *something’s happening to me’*. *And I do understand that with the resources at the hospital and you see a young guy that*, *apart from explaining himself badly*, *so well*, *nothing’s happening to this guy*. *I understand*, *but if he* [service user] *is insisting*… *you know*? *And well*, *they found that I had Helicobacter pylori*. *And I said ‘see*, *dad*, *you said that I did not have anything*, *and no*, *and that I am annoying*, *and so on*…*’ but well (*…*) Ufff*… *I ended up fed up* [of insisting]*”*. *(P27*, *man*, *28 years old*).

Most participants valued how healthcare professionals communicated and related to service users over medical skills and experience. Empathy, active listening, warmth and shared-decision making (i.e., core elements of horizontal relationships) were highly valued by participants, although they also mentioned that at least some experience and good praxis were essential. This was especially relevant for primary healthcare professionals. For secondary healthcare professionals, expertise seemed to be more valued for some as health conditions treated within secondary care were commonly more severe and urgent.

*“Not the experience itself* [is important], *because many times*, *people who have just finished university or that doesn’t have much experience are more attentive or they do it better*, *compared to someone who already has a lot of experience*, *and they’ve seen everything… “what are you going to say to me*?*”*, *sometimes experience is the contrary of what it should be*.*” (P18*, *man*, *60 years old*).

It is important to highlight participants’ experiences of stigmatisation and discrimination from healthcare professionals. It was not uncommon participants had felt judged for how they experienced their health, sociodemographic characteristics or elements of their identity.

*“I don’t know… if he* [the doctor] *was racists or it was how he is*, *I don’t want to judge him… but I came in and I told the doctor*, *he was writing… “yes*, *what do you want*?*”*, *in a despotic manner*. *I left crying*, *I remember*, *I left crying because I didn’t like it*. *I felt humiliated… […] and at that time I was going through depression […] he stand up*, *only stretch his… his head*, *he made me put my foot up*, *he didn’t even came close to look at it*, *nothing*. *“Ah*, *that is dryness*, *put on any hydrating cream*.*” (P29*, *woman*, *55 years old*).

In the information needed from healthcare professionals, participants mentioned how they did not need technical or scientific details, but information around their health condition in a comprehensible and clear language. It was important that information on the potential causes (or triggers) of an ARLTI were shared, and any other information if the service user requested it.

*“I am curious*, *I like… to know things and why… I would like for them to explain to me ‘look*, *the cough is coming for this reason*, *or for the other*, *because the flu attacks here or attacks there’*, *I would have liked this*, *of course… I would go out* [from the healthcare centre] *more convinced if they explain things to be […] If I understand*, *then… I’ll take it* [medication]*”*. *(P1*, *man*, *75 years old*).

## Discussion

One of the core elements highlighted across all themes identified was the prevalent tension among service users between the need for dependency and reliance (on healthcare professionals and the health system) and, for some, the need for autonomy to care for one’s health. The discussion around where power in healthcare lays is not new [67–71]. As previous research has identified, healthcare systems are strongly grounded on the biomedical model, and capitalism as the overarching system. These two models have strongly shaped healthcare and have led to the marketization of people’s health, so that the human body is understood and treated a “machine to repair” rather than a system that needs to be understood in its whole subjective complexity [69–71].

As portrayed in this piece of research, power struggles within healthcare services appeared to create strains in the relationships, not only between service users and health professionals but also between users and the healthcare system. These strains were often expressed through the questioning of trust towards health professionals and “the system”. In turn, these could be translating into non-adherence to medication and “the transgression” of some stablished rules within healthcare systems (e.g., attending emergency services *only* for what was considered urgent *for* health professionals). This is where the importance for AMR lays, in understanding that health system users’ might need to become active agents of their health, with the support of health professionals and health systems.

As already discussed in the literature [72–74], a concordance approach to prescribing could be helpful to engage and support service users into making shared decisions over medicines use. However, inherent power imbalances in healthcare consultations may still hinder service users’ attempts to exercise their power when making decisions over antibiotic prescribing. Also, as previous research has highlighted, efforts may need to be re-directed towards accepting service users’ resistance to medicines and develop safer medical treatments [72].

This argument is consistent with acknowledging that the “bodies” in healthcare are owned by the people who carry them, and are not the property of health professionals and healthcare systems [67]. Integrating these ideas and values into healthcare systems would mean a shift in the understanding of “health” and the role of Medicine and other health professions. This model of health would place health professionals in a supporting and guiding role, instead of keeping them in a dogmatic and authoritative one. This should however not be seen as to downplay the expertise of health professionals, or to compromise adherence to clinical guidelines [75], but to place health professionals into a position that could allow service users to take agency over their own health and to promote body awareness and health literacy [76–78]. Allowing service users to become subjective and reliable experts of their bodies, as well as active agents of their health, could ease the powerlessness and uncertainty that some service users experience towards improving their health. These findings are consistent with previous research on the need to improve health professionals-service users’ communication [26–29, 79], to foster two-way communication in clinical consultations [74], and to promote shared-decision making [20, 30, 31, 80–83] to improve health outcomes and reduce antibiotic prescribing [30, 83]. Based on the concept of *translation* in sociology [84], Efforts to improve communication within healthcare should also consider the variance in the languages used by different agents in healthcare (e.g., service users and doctors) [79].

However, power imbalances need to be addressed structurally to enable shared-decision making [85]. Our suggestion would be to start with promoting structural changes within healthcare systems that could allow health professionals to apply alternative approaches within healthcare consultations. Also, to encourage healthcare professionals to adopt new perspectives to healthcare at a local/community level. For instance, co-developing (with service users) and applying protocols for antibiotic prescribing/use in which power in clinical decisions is shared through active listening and the promotion of service users’ agency. Other examples could be to train and encourage healthcare professionals to help service users to voice their expectations and agendas for healthcare consultations [86]. Nursing professionals are actually in-between power struggles in healthcare, as they might often be perceived as “less capable” than professionals of medicine, by both service users and other health professionals. Nurses could however have an important role in promoting a model of healthcare that encourages agency among users and challenges the medicalisation and marketization of health.

It is also worth discussing that not all service users seemed to be ready and/or willing to embody a position of power over their health [87]. Individual characteristics aside, this is intrinsic to the historical place of authority that Medicine and health sciences have occupied [68, 69, 76–78]. Shifting these roles is rather a sociological and cultural challenge that cannot be undermined neither ignored. It again means to convulse the foundations of current and historical systems of power embedded, not only within healthcare systems, but political and economic institutions. It should be noted that more subtle ways of agency among service users (e.g., disclosing medical misdeeds [87] or resistance to treatment recommendations [88]) need to be acknowledged as agentic behaviours and not be misinterpreted. However, claims of agency in research interviews may not necessarily match service users’ actual demands within healthcare consultations [86].

Service users’ expectations, especially those in situations of social vulnerability (e.g., women or the elderly) are inevitably based on recurrent experiences of vertical relationships and social disempowerment [69]. Thus, it is not surprising that people’s expectations in these cases are generally to become passive subjects that need health professionals and health systems to take control of their health. Interestingly, some of those participants who had integrated a role of passiveness towards their health sometimes challenged health professionals’ prescriptions. We could argue that this hints that some “patients” may be willing but not able (or ready) to fully become active agents (yet). This resistance to medical authority may be as well a way to gain control and power over their health [88].

On the other hand, some participants seemed to not question (neither directly nor indirectly) the role of power of health professionals. Maintaining a passive role could provide them with certainty, placing the responsibility of caring for their health externally. The potential intersection of social vulnerabilities (e.g., migration or low socio-economic status) among some participants in this study should be noted [55]. Despite data were not revealing enough of the intersection of inequities, the potential experiences of marginalisation need to be considered to understand some of the participants’ accounts. Further research should focus on vulnerable and socially excluded communities to explore agency in health and the relationships with health professionals and the healthcare system. Intersectional approaches to social and health inequities could be highly relevant.

It is also important to be conscious of the vulnerable position people are when they attend healthcare services, as people are often either ill or concerned about their health. Service users expect to get answers to their questions as a way to ameliorate their concerns and get a sense of control over their health. In the context of ARLTIs, service users expected to get solutions to their symptoms, and antibiotics seemed to be perceived as to provide the sense of control service users were seeking. The need for active listening and interactions based on empathy and respect for service users’ subjective experiences need to be central to healthcare consultations. Service users should not only be “at the centre” of healthcare consultations, but of their overall health. Based on participants’ accounts in this study, healthcare interactions based on active listening, empathy and respect for service users’ subjective experiences may be central to encourage service users to care for their health. Taking these actions could inevitably contribute to a more reasonable use of antibiotics among service users, by supporting health literacy and body awareness. In turn, these changes could promote service users’ satisfaction and expectations towards healthcare consultations, important factors for the use and prescription of antibiotics [2, 21, 22]. Also, balancing power in healthcare could promote the use of delayed prescriptions for antibiotics.

Health information materials could also be useful to improve, not only health literacy but body and health awareness among service users. These should however not be developed without the involvement of the community. Patient and Participant Involvement (PPI) [89, 90] and Responsible Research & Innovation (RRI) [91] principles need to then be considered in research so that research impact can be more in-line with communities’ needs and preferences.

Moreover, service users’ views of time in relation to health (e.g., illness progression) and healthcare (e.g., healthcare consultation length) seemed to be well linked to a consumerist approach to health. The prevalence of this approach translates into service users often seeking “quick fixes” to regain their health (and lifestyles) [39, 55]. These are also expected to be provided by health professionals and sought in private healthcare. As the accounts in this research have shown, antibiotics were perceived to be one of the “quick fixes” for ARLTIs. Thus, despite service users could be aware of the dangers of overusing (and misusing) antibiotics, they were still often persuaded by the perceived high effectiveness and efficiency of antibiotics. We then need to reflect on the relevance of merely increasing AMR knowledge and awareness, as the influence of socio-structural factors (e.g., the marketization of health) may be limiting the impact of knowledge and awareness on antibiotic use [39].

Spanish public healthcare has suffered from intense financial cuts in the last decade [92], and it is increasingly pressured due to the current COVID-19 pandemic [93]. It will be important to pay attention to the potentiality of an increased number of people accessing private services in response to the deterioration of public healthcare. This can be a concern for antibiotic overuse, as participants in this study expressed that private healthcare was a good resource for antibiotic prescriptions, compared to public healthcare. This variances in antibiotic prescribing between public and private healthcare systems has already been described in the literature [94].

Consistently with previous research, we can conclude that this research highlights the need for a more humanistic and service users’-centred approach to health to face the challenges of AMR [95], based on shared power and service users’ autonomy in healthcare [30]. This should however be accompanied by professionals’ training on how to share power and promote horizontal communication and relationships with service users [96]. Besides, it is important to consider structural limitations and other barriers already identified in previous research, such as time constraints in primary healthcare consultations [97] and limitations that healthcare professionals encounter to negotiate antibiotic prescribing decisions with service users [98].

## Conclusions

Power dynamics and autonomy in healthcare need to be considered as important factors to promote a responsible use of antibiotics, and to prevent AMR. Changing power dynamics and shifting service users’ role in healthcare is however challenging. Future research should include the perspectives of healthcare professionals and other key actors (e.g., policymakers), to better understand the impact of power dynamics and autonomy not only on antibiotic use but antibiotic prescribing. Findings from this research can inform the development of policies and protocols to prevent AMR, clinical practice and future research.

## Supporting information

S1 File Interview topic guide (Spanish). (DOCX)

S2 File Interview topic guide (English). (DOCX)
