# Supplementary material for: Autonomy, power dynamics and antibiotic use in primary healthcare: A qualitative study
Source: PLoS One. 2020 Dec 18;15(12):e0244432. doi: 10.1371/journal.pone.0244432 (PMC7748265; doi:10.1371/journal.pone.0244432)
Supplement: S1 File — (DOCX) [file pone.0244432.s001.docx]

**S1 File. Topic guide (Spanish)**

| ***Por favor, querríamos saber sus opiniones y experiencias sobre:*** | |
| --- | --- |
| **Experiencias con las infecciones respiratorias agudas** | *1a. ¿Cuáles han sido sus experiencias con un [resfriado de pecho]?*  *2a. ¿Cómo se han visto afectadas las actividades diarias durante estas experiencias?*  *3a. ¿Cómo más cree usted que le puede haber afectado tener un [resfriado de pecho]?*  *4a. ¿Qué entiende usted por una infección?*  *5a. ¿Qué es lo que entiende usted por una bronquitis?* |
| **Descripción de síntomas y qué palabras utiliza** | *1a. ¿Me podría explicar qué síntomas tuvo cuando tuvo el [resfriado de pecho]?  2a. ¿Me podría describir, con sus palabras, los síntomas del [resfriado de pecho] que conoce?*  *3a. ¿Qué cree que le lleva a tener un [resfriado de pecho]?* |
| **Signos de alarma para consultar con servicios médicos** | *1a. ¿Qué síntomas le hacen pensar que podria tener un [resfriado de pecho]?*  *2a. ¿Qué le hace pedir visita con un profesional de la salud para un [resfriado de pecho]?*  *3a. ¿Alguna vez que ya se ha visitado, qué le hace volver a consultar?* |
| **Servicios que atiende para las infecciones respiratorias agudas** | *1a. ¿A dónde se dirige cuando tiene un [resfriado de pecho]?*  *2a. ¿Cuál fue su experiencia yendo a [donde se haya dirigido]?*  *3a. ¿Qué información recibió?*  *4a. ¿Cuál cree que es el tiempo necesario para una consulta para un [resfriado de pecho] con un profesional sanitario?* |
| **Experiencias con los tratamientos recibidos** | *1a. ¿Qué tratamientos ha utilizado has ahora para un [resfriado de pecho]?*  *2a. ¿Alguna vez ha tenido efectos secundarios a algún tratamiento?*  *3a. ¿Cómo fue su experiencia utilizando el tratamiento en casa?* |
| **Preferencias y tipos de tratamiento** | *1a. ¿cuál cree que es el mejor tratamiento para los [resfriados de pecho]?*  *2a. ¿Podría explicar qué tratamiento, con o sin medicamentos, ha recibido durante la visita?*  *3a. ¿Qué tratamiento, con o sin medicamentos, esperaba recibir?*  *4a. ¿Me podría explicar si pudo decidir el tratamiento conjuntamente con el médico/enfermero?*  *5a. ¿Cómo cree que le puede ayudar tomar antibióticos para un [resfriado de pecho]?*  *6a. ¿Alguna vez ha tenido la necesidad de tomar antibióticos, aunque no se los haya recetado su médico?*  *7a. ¿Le han dado alguna vez una receta para antibióticos que sólo podía utilizar cuando no mejoraba en unos días?* |
| **Conocimiento resistencia antibióticos** | *1a. Si se ha tomado antibióticos alguna vez, ¿qué pautas ha seguido para tomarlos?*  *2a. ¿Qué motivos le llevan a dejar de tomar antibióticos antes de completar el tratamiento?*  *3a. ¿Qué cree que es importante de completar el tratamiento con antibióticos?* |
| **Habilidades de comunicación profesionales de la salud** | *1a. ¿Cómo describiría su relación con su médico?*  *2a. ¿Con qué profesional sanitario prefiere visitarse?*  *3a. ¿Me podría explicar si ha tenido una mala experiencia/experiencia desagradable con un profesional sanitario alguna vez?*  *4a. Cuando ha recibido tratamiento para un [resfriado de pecho], ¿cuál fue su experiencia respecto a la información recibida sobre el tipo de tratamiento?*  *5a. Cuando el profesional de la salud le explica sobre qué le pasa y el tratamiento, ¿cree que sus indicaciones son claras?*  *6a. ¿Qué es lo que le gusta de la consulta con el profesional sanitario?*  *7a. Algunas de las personas con las que hemos hablado nos han comentado que alguna vez han descrito los síntomas a su médico/enfermero como si fuesen más graves de lo que son para que la enfermera/médica le recetase un tratamiento que les funcionase mejor. ¿A usted le ha pasado esto alguna vez?*  *8a. ¿Me podría explicar si cree que tiene la oportunidad de hacer preguntas libremente al profesional sanitario?* |

**Para finalizar**• ¿Qué le parece su participación en este proyecto?
• En vuestra opinión, ¿en qué os gustaría ayudar?
**Finalización de la entrevista**
• Resumen breve de los puntos claves de la entrevista
• Preguntar si quiere añadir algo más
• Agradecimientos
• Recordatorio de utilidad de los resultados y aspectos éticos
