# Supplementary material for: Autonomy, power dynamics and antibiotic use in primary healthcare: A qualitative study
Source: PLoS One. 2020 Dec 18;15(12):e0244432. doi: 10.1371/journal.pone.0244432 (PMC7748265; doi:10.1371/journal.pone.0244432)
Supplement: S2 File — (DOCX) [file pone.0244432.s002.docx]

**S2 File. Topic guide (English)**

| ***Please, we would like to know your opinion and experiences on:*** | |
| --- | --- |
| **Experiences with acute lower respiratory tract infections** | *1a. What are your experiences of an ALRTI?*  *2a. How have your daily activities been impacted due to experiencing ALRTIs?*  *3a. How else do you think that having an ALRTI has had an impact on you?*  *4a. What do you understand by an infection?*  *5a. What do you think a bronchitis is?* |
| **Symptom description and used terminology** | *1a. Could you tell me about the ALRTI symptoms that you experienced?  2a. Could you describe, with your own words, the ALRTI symptoms that you know about?  3a. What do you think are the risk factors for an ALRTI?* |
| **Red flag symptoms to seek professional assistance** | *1a. What symptoms make you think that you could have an ARLTI?*  *2a. What is a trigger for you to visit a healthcare professional for an ALRTI?*  *3a. What makes you re-visit a healthcare professional?* |
| **Access to health services for ALRTIs** | *1a. What services do you access for an ALRTI?*  *2a. What was your experience attending [service accessed]?*  *3a. What information did you receive?*  *4a. What is the appropriate duration of a consultation with your healthcare professional for an ALRTI?* |
| **Experiences with treatments received** | *1a. What treatments have you used for an ALRTI?*  *2a. Have you ever experienced side effects from any treatment?  3a. How was your experience using this treatment at home?* |
| **Preferences and type of treatments** | *1a. What do you think is the best treatment for ARLTIs?*  *2a. What treatments have you received in your visit to healthcare professionals?*  *3a. What treatment were you expecting to receive?*  *4a. Could you decide, together with the healthcare professional, what was the best treatment for you?*  *5a. How do you think antibiotics could help for ALRTIs?*  *6a. Have you ever had the need to take antibiotics, even if your doctor had not prescribed them?*  *7a. Have you ever gotten a prescription that you could only use if you were not getting better in a few days?* |
| **Knowledge on antibiotic resistance** | *1a. If you have taken antibiotics before, what guidelines have you followed to use them?*  *2a. In your experience, what are the reasons to stop using antibiotics before completing the treatment?*  *3a. What do you think is important of completing a treatment with antibiotics?* |
| **Healthcare professionals’ communication skills** | *1a. How would you describe your relationship with your healthcare professional?*  *2a. Which healthcare professional do you prefer to get a visit with?*  *3a. Have you ever had a negative experience with a healthcare professional?*  *4a. What was your experience in relation to the information received by healthcare professionals when you received treatment for an ALRTI?*  *5a. When a healthcare professional tells you about the diagnosis and treatment, do you think they communicate clearly?*  *6a. What do you like of your consultation with your healthcare professional?*  *7a. Some of the people that we have spoken to have mentioned that they have sometimes described their symptoms to a healthcare professional as if they were more severe than they were to receive more effective treatment. Has this ever happened to you?*  *8a. Do you think you have the opportunity to ask questions freely to your healthcare professional?* |

**Before the end of the interview**

• What do you think of your participation in this study?
• In your opinion, what would you like to contribute on?

**End of the interview**
• Brief summary of main points discussed in the interview
• Ask if they want to add anything else
• Thank you
• Reminder about the use of the findings and ethical aspects
